# DNA viruses in human cancer: established oncogenic mechanisms, emerging virus–tumour associations and clinical implications

**DOI:** 10.3389/fcimb.2026.1970942

**Published:** 2026-10-26

**Authors:** Zhen Liu, Yan Wang, Ran Liu, Yuan-He Wang, Peng-Yuan Su, Zhi-Chao Han, Shuo Yuan, Bin-Hai Yu, Cheng-Ye Che, Shao-Qi Tian

**Affiliations:** 1 Department of Orthopedic Surgery, the Affiliated Hospital of Qingdao University, Qingdao, China; 2 Department of Gynecology, The Women and Children’s Hospital of Qingdao, Qingdao, China; 3 Department of Pediatrics, Peking University Third Hospital, Beijing, China; 4 Department of Ophthalmology, the Affiliated Hospital of Qingdao University, Qingdao, China

**Keywords:** DNA viruses, molecular diagnosis, viral integration, viral oncogenesis, virus-associated cancers, virus–host interactions

## Abstract

DNA virus-associated tumours represent an important component of the global burden of infection-associated cancer. Studies of classical DNA tumour viruses have defined major oncogenic routes, including persistent viral oncoprotein expression, chronic inflammation and tissue regeneration, viral integration and immune evasion. This work has also facilitated clinical applications of vaccines, antiviral therapies and viral biomarker testing. Recent advances in cancer genomics and molecular pathology have identified several biologically distinctive virus–tumour associations. These include TTMV::RARA fusion-driven APL-like acute myeloid leukaemia, HPV42-associated digital papillary adenocarcinoma, AAV2 insertions in a subset of hepatocellular carcinomas and transplant-associated BKPyV-positive urothelial carcinoma. The significance of these associations lies not in expanding the catalogue of tumour viruses, but in revealing distinct molecular events. These encompass virus–host fusion, tissue-specific viral oncogene expression, insertion-mediated cis activation and persistent T-antigen expression under immunosuppression. Here, we outline the principal mechanisms of classical DNA tumour viruses and compare the molecular basis, modes of tumour maintenance and potential clinical value of these recently recognised associations. We also discuss detection strategies, evidentiary boundaries and priorities for future research. Current evidence indicates that event-matched assays may support specialist ancillary diagnosis or molecular classification in selected settings, particularly for APL-like leukaemia and DPA. However, none of the four emerging associations currently supports population screening or a virus-directed standard therapy, and research-stage applications require prospective validation.

## 1 Introduction

Infectious agents are important contributors to the global burden of cancer, and some infection-associated cancers can be prevented through vaccination, treatment of the underlying infection, and screening. In 2018, an estimated 2.2 million new cancer cases worldwide were attributable to infectious agents, accounting for approximately 13% of all incident cancers (de Martel et al., 2020). Cancers associated with human papillomavirus (HPV), hepatitis B virus (HBV) and Epstein–Barr virus (EBV) represented a substantial proportion of this burden. In 1964, Epstein, Achong and Barr identified EBV in cells derived from Burkitt lymphoma, marking the discovery of the first human tumour virus (Epstein et al., 1964). Subsequent research on tumour viruses not only established links between several viruses and specific cancers but also helped shape fundamental concepts in cancer biology, including oncogenes, tumour suppressor genes and cell-cycle regulation. Vaccination, interruption of transmission and antiviral therapy can reduce the burden of certain viral infections and the subsequent risk of cancer (Chang et al., 2009; Falcaro et al., 2021), thereby linking tumour virology directly to cancer prevention.

The principal human oncogenic DNA viruses recognised to date include high-risk HPV types, HBV, EBV, Kaposi sarcoma-associated herpesvirus (KSHV) and Merkel cell polyomavirus (MCPyV) (Raab-Traub and Flynn, 1986; Chang et al., 1994; Walboomers et al., 1999; Chen et al., 2006; Feng et al., 2008). These viruses differ markedly in genome organisation, tissue tropism and life cycle, yet each can contribute to tumour development through persistent viral protein expression, disruption of cell-cycle control and the DNA-damage response, reprogramming of host transcription and the immune microenvironment, chronic tissue injury, or selected integration events (Zapatka et al., 2020). Research on these established associations has enabled vaccine-based prevention, viral biomarker testing and selected approaches to disease monitoring and treatment (Chang et al., 1997; Lei et al., 2020).

In this Review, ‘oncogenic DNA virus’ denotes a virus with established causal evidence for one or more human cancers, whereas ‘virus-associated tumour’ describes a tumour in which a viral signal or virus–host event has been reproducibly reported without implying an equivalent level of causality.

Advances in metagenomic and transcriptomic sequencing, viral capture sequencing and long-read analysis are shifting the focus from asking whether a tumour harbours a particular virus to determining which virus–host molecular events are retained in tumour cells (Zapatka et al., 2020). Against this backdrop, several associations have recently been recognised, including acute promyelocytic leukaemia (APL)-like acute myeloid leukaemia driven by the TTMV::RARA fusion, HPV42-associated digital papillary adenocarcinoma, adeno-associated virus type 2 (AAV2) insertions in a small subset of hepatocellular carcinomas, and transplant-associated BK polyomavirus (BKPyV)-positive urothelial carcinoma (Nault et al., 2015; Astolfi et al., 2021; Leiendecker et al., 2023; Starrett et al., 2023). Rather than representing a single new mode of oncogenesis, these associations involve distinct molecular mechanisms, including virus–host fusions, tissue-specific expression of viral oncogenes, low-frequency insertional activation and persistent T-antigen expression in the context of immunosuppression. In this Review, we first outline established human oncogenic DNA viruses and their principal mechanisms, then compare the molecular basis, implications for tumour biology and potential clinical relevance of these recently recognised DNA virus–cancer associations. We also discuss detection strategies, the limits of current evidence and directions for future research.

### 1.1 Literature search and selection

This narrative Review was informed by a structured search of PubMed from the earliest indexed records available through 27 September 2026. The search combined virus-specific terms (high-risk HPV, HBV, EBV, KSHV or HHV-8, MCPyV, TTMV or torque teno mini virus, HPV42, AAV2 or adeno-associated virus 2, and BKPyV or BK polyomavirus) with tumour-related terms (cancer, carcinoma, tumour or tumor, neoplasm, leukaemia or leukemia, lymphoma, integration, fusion, oncogenesis and viral antigen) using Boolean operators. For the four emerging associations, additional searches combined the relevant viral or virus–host event with the corresponding disease terms, including TTMV::RARA and APL-like acute myeloid leukaemia, HPV42 and digital papillary adenocarcinoma, AAV2 insertion and hepatocellular carcinoma, and BKPyV and transplant-associated urothelial carcinoma. Because this was a narrative rather than a systematic review, we did not undertake formal study-quality scoring or meta-analysis.

## 2 Established human oncogenic DNA viruses

High-risk HPV types, HBV, EBV, KSHV and MCPyV exemplify several major routes through which DNA viruses contribute to human cancer. These include persistent viral oncoprotein expression, latency-associated gene programmes, chronic inflammation and tissue regeneration, viral integration and immune evasion. **Table 1** summarised their associated malignancies, key viral factors, principal mechanisms and established clinical implications, and the sections below consider each virus in turn.

**Table 1:** Principal mechanisms and clinical relevance of five human oncogenic DNA viruses.

Virus	Major associated tumours	Key viral factors	Principal mechanisms	Clinical relevance
High-risk HPV	Cervical cancer, other anogenital cancers and a subset of oropharyngeal cancers	E5, E6 and E7	E6/E7 disrupt p53 and pRB–E2F signalling, activate telomerase and increase replication stress; E5 contributes to growth signalling and immune evasion	Established: prophylactic vaccination, cervical screening, and site-specific HPV- or p16-based tumour classification. Promising or investigational: circulating tumour HPV DNA and therapeutic vaccination in defined settings.
HBV	Hepatocellular carcinoma	HBx and aberrant preS/S surface proteins	Chronic inflammation and compensatory regeneration; viral protein-mediated signalling and cellular stress; integration-associated structural variation or dysregulation of nearby genes	Established: vaccination, prevention of mother-to-child transmission, antiviral treatment of chronic HBV infection, and HCC surveillance in at-risk patients.
EBV	Nasopharyngeal carcinoma, EBV-positive gastric cancer and multiple lymphoma subtypes	EBNA1, EBNA2/3, LMP1/2, EBER and BART miRNAs	Episome maintenance; promotion of proliferation and resistance to apoptosis; remodelling of host transcription, the epigenome and immune microenvironment	Established or specialist: EBER in situ hybridisation for tumour classification and plasma EBV DNA monitoring in nasopharyngeal carcinoma. Context-dependent: EBV-directed cellular therapy in selected post-transplant lymphoproliferative disease settings.
KSHV	Kaposi sarcoma and primary effusion lymphoma	LANA, vCyclin, vFLIP, vGPCR and vIL-6	Latency maintenance and cell-cycle progression; NF-κB activation; promotion of angiogenesis and inflammatory paracrine signalling	Established: LANA immunohistochemistry, HIV control and immune-status management, with tumour-directed treatment according to disease and stage.
MCPyV	Merkel cell carcinoma	Truncated LT and sT	Truncated LT disrupts pRB–E2F control; sT remodels transcription through MYCL–MAX–EP400; established tumours remain dependent on T-antigen expression	Established: T-antigen immunohistochemistry and viral nucleic-acid testing support MCC classification; treatment is selected by stage. Research: antigen-directed vaccines and virus-specific T-cell approaches.

### 2.1 High-risk HPV

High-risk HPVs infect stratified squamous epithelia and cause cervical and anal cancers, subsets of vulvar, vaginal and penile cancers, and a substantial fraction of oropharyngeal cancers. Tumour development is linked primarily to persistent infection and continued E6/E7 expression rather than to transient viral detection (Rodriguez et al., 2008; Walboomers et al., 1999).

During early infection, HPV genomes are generally maintained as extrachromosomal episomes (The Cancer Genome Atlas Research Network, 2017). As lesions progress, some viral genomes integrate into host chromosomes, with breakpoints frequently affecting the E1 or E2 regions. This can relieve E2-mediated repression of the early promoter and promote E6/E7 expression. Integration may also be accompanied by local copy-number amplification, chromosomal rearrangements and the formation of virus–host fusion transcripts (Akagi et al., 2014; Parfenov et al., 2014). However, integration does not occur in all HPV-associated tumours (The Cancer Genome Atlas Research Network, 2017). In tumours in which episomal HPV predominates, E6/E7 expression can also be maintained through methylation of the long control region, chromatin remodelling and altered E2 regulation.

E6 and E7 target the p53 and pRB tumour-suppressor pathways, respectively (Munger et al., 1989; Scheffner et al., 1990). E6 forms a complex with the host E3 ubiquitin ligase E6AP, promoting p53 degradation, and can enhance TERT transcription and telomerase activity (Klingelhutz et al., 1996). E7 binds pRB-family proteins and releases E2F, enabling differentiating epithelial cells to re-enter S phase (Munger et al., 1989). Together, E6 and E7 promote replication stress, centrosome abnormalities and chromosomal instability (Goodwin et al., 2000). Viral replication also engages ATM- and ATR-associated DNA-damage responses, while infection-associated APOBEC activity can contribute to host-genome alterations (Moody and Laimins, 2009; The Cancer Genome Atlas Research Network, 2017).

HPV can also reduce antigen presentation and disrupt interferon signalling, thereby weakening local immune clearance (Ashrafi et al., 2005). After tumour development, most HPV-driven tumours continue to express E6/E7. Inhibiting E6/E7 can reactivate the p53 and pRB pathways and induce cell-cycle arrest, senescence or apoptosis. Thus, E6 and E7 contribute both to malignant transformation and to the continued proliferation and survival of HPV-positive tumour cells (Goodwin et al., 2000). Accordingly, both oncoproteins are major targets in the development of virus-specific therapies.

### 2.2 HBV

HBV-associated hepatocarcinogenesis is shaped by persistent cccDNA-supported transcription, chronic liver injury, viral protein activity and selected integration events. After entry into hepatocytes, relaxed circular DNA is repaired to form cccDNA, a nuclear minichromosome that can sustain long-term viral transcription and relapse after treatment withdrawal (Tuttleman et al., 1986).

HBV-associated hepatocellular carcinoma (HCC) typically develops in the context of longstanding inflammation, tissue damage and clonal evolution (Chen et al., 2006). Liver injury arises mainly from sustained host immune responses against infected hepatocytes. Repeated cycles of cell death and compensatory regeneration, compounded by oxidative stress, inflammation and fibrosis, allow DNA damage and replication errors to accumulate (Chen et al., 2006). Cirrhosis is an important risk context, but HCC can also develop in some patients without cirrhosis (Sung et al., 2012). This suggests that viral proteins and integration events may make additional pro-oncogenic contributions.

HBV X protein (HBx) forms a complex with DDB1 that promotes degradation of the SMC5/6 complex, thereby relieving SMC5/6-mediated restriction of cccDNA transcription (Decorsiere et al., 2016; Murphy et al., 2016). This host restriction axis directly links persistent viral transcription to HBx function. Deletions or mutations in the preS/S region during chronic infection can cause HBV surface proteins to accumulate in the endoplasmic reticulum. This accumulation triggers the unfolded protein response and oxidative stress (Liu et al., 2022). Together with HBx and host genomic alterations, these effects contribute to HCC development.

Double-stranded linear DNA generated during HBV replication can integrate at diverse sites in the host genome (Sung et al., 2012). Most integration events confer no clear selective advantage, whereas a minority become enriched in tumours through the clonal expansion of abnormal hepatocytes. HBV integration can generate deletions, amplifications and chromosomal rearrangements, as well as virus–host fusion transcripts. Integration events recurrently involve loci such as TERT, KMT2B and CCNE1 (Sung et al., 2012; Peneau et al., 2022). Some integrated fragments can also direct the expression of truncated HBx or aberrant surface proteins. Collectively, chronic tissue damage, viral protein activity and genomic integration form the biological basis of HBV-associated HCC.

### 2.3 EBV

EBV establishes lifelong episomal latency in B lymphocytes and epithelial cells. It is causally associated with nasopharyngeal carcinoma, EBV-positive gastric cancer, Burkitt lymphoma, classic Hodgkin lymphoma, extranodal NK/T-cell lymphoma and immunodeficiency-associated lymphoproliferative disorders (Raab-Traub and Flynn, 1986; Weiss et al., 1987; Shibata and Weiss, 1992).

Viral gene expression in EBV-associated tumours follows distinct latency programmes shaped by cell lineage and immune selection pressures (Rowe et al., 1987; Young et al., 1989). Burkitt lymphoma generally exhibits latency I, with expression largely restricted to EBNA1, EBV-encoded small RNAs (EBERs) and BART miRNAs (Rowe et al., 1987). Nasopharyngeal carcinoma, classic Hodgkin lymphoma and extranodal NK/T-cell lymphoma typically exhibit latency II, which can include LMP1 and LMP2 expression (Pallesen et al., 1991). Immunodeficiency-associated lesions, including post-transplant lymphoproliferative disease, can exhibit latency III, with broader expression of EBNA and LMP proteins (Young et al., 1989). EBV-positive gastric cancer generally maintains a more restricted latency programme (Sugiura et al., 1996).

EBNA1 binds the viral origin of plasmid replication (oriP) and tethers episomes to host chromatin, enabling their stable replication and segregation during cell division (Yates et al., 1984). LMP1 mimics constitutively active CD40 signalling and activates pathways including NF-κB (Mosialos et al., 1995; Mitra et al., 2023). LMP2A rewires B-cell receptor signalling and maintains PI3K/AKT activity (Caldwell et al., 1998; Mancao and Hammerschmidt, 2007). In latency III, EBNA2 and the EBNA3 family further alter host transcriptional programmes involved in cell growth, cell-cycle regulation and apoptosis (Young et al., 1989).

EBV latent proteins can also remodel the cellular epigenetic state through host transcription factors, DNA methyltransferases and chromatin-regulatory complexes. EBERs and BART miRNAs regulate cell survival, antigen presentation and the expression of immune checkpoint-related genes (Nachmani et al., 2009; Marquitz et al., 2012). Latent proteins and the extracellular vesicles they regulate can also influence neighbouring cells through paracrine signalling (Nkosi et al., 2020). Together, episome maintenance, restricted latent gene expression, epigenetic remodelling and immune evasion support the sustained growth of EBV-associated tumours (Marquitz et al., 2012; Mitra et al., 2023).

### 2.4 KSHV

KSHV establishes predominantly latent infection in endothelial cells and B lymphocytes and causes Kaposi sarcoma and primary effusion lymphoma; it is also associated with KSHV-associated multicentric Castleman disease. HIV infection, transplantation and other immunosuppressive states substantially increase disease risk (Chang et al., 1994; Cesarman et al., 1995; Soulier et al., 1995).

Most tumour cells in KSHV-associated malignancies maintain latent infection. Latency-associated nuclear antigen (LANA) tethers circular viral episomes to host chromatin (Ballestas et al., 1999) and can interfere with the p53 and pRB/E2F pathways (Friborg et al., 1999). The viral cyclin (vCyclin) forms an active complex with CDK6 and promotes cell-cycle progression (Godden-Kent et al., 1997). vFLIP persistently activates NF-κB through the IKK complex, enhancing the expression of anti-apoptotic and inflammatory genes (Field et al., 2003; Bagneris et al., 2022). Other viral proteins and miRNAs can also modulate growth signalling and interfere with interferon responses and antigen presentation. These activities enable infected cells to survive and expand without producing large numbers of virions.

A small fraction of cells within KSHV-associated lesions also enter the lytic programme. vGPCR, vIL-6 and multiple chemokines expressed during the lytic phase can promote angiogenesis, inflammatory-cell recruitment and proliferation of neighbouring cells (Bais et al., 2003; Rivera-Soto et al., 2020). Latently infected cells form the bulk of the lesion, whereas lytically infected cells provide paracrine support. At the same time, immunosuppression weakens host control of infected cells (Ballestas et al., 1999; Rivera-Soto et al., 2020). Together, these three components shape the growth environment of KSHV-associated tumours. The regression of some Kaposi sarcomas after immune reconstitution further indicates that viral programmes and host immune status jointly influence lesion progression.

### 2.5 MCPyV

MCPyV-positive Merkel cell carcinoma (MCC) is defined by persistent viral T-antigen expression, whereas MCPyV-negative MCC usually carries a prominent ultraviolet-damage signature and a higher somatic mutation burden (Feng et al., 2008; Harms et al., 2015; Wong et al., 2015; Vaidya et al., 2025).

MCPyV-positive MCC is characterised by two coordinated alterations of the viral genome. First, viral DNA undergoes clonal integration into the host genome (Feng et al., 2008). Second, the large T antigen (LT) encoded by the integrated virus carries tumour-specific premature stop or frameshift mutations (Shuda et al., 2008). Truncated LT loses its C-terminal helicase and replication-initiation domains and can no longer support viral DNA replication. However, it retains the N-terminal LXCXE motif and the ability to bind pRB (Shuda et al., 2008), sustaining cell-cycle progression while lytic replication is disabled.

The small T antigen (sT) cooperates with truncated LT to maintain MCPyV-positive MCC. sT promotes the association of MYCL–MAX with the EP400 chromatin-regulatory complex (Cheng et al., 2017), thereby remodelling transcriptional programmes related to cell survival, metabolism and tumour phenotype. Experimental suppression of LT and sT can induce arrest or death in MCPyV-positive MCC cells (Houben et al., 2010), indicating that established tumours remain dependent on T-antigen expression. In 2025, the International Agency for Research on Cancer classified MCPyV as carcinogenic to humans (Group 1) and concluded that it causes MCC, further establishing this virus–tumour association (Karagas et al., 2025).

Across the five established oncogenic DNA viruses, recurrent mechanisms include disruption of p53 or pRB–E2F control, chronic inflammation and compensatory regeneration, episomal latency or clonal integration, immune evasion, epigenetic or transcriptional reprogramming, and induction or exploitation of DNA-damage responses. The relative contribution of each mechanism differs by virus and tumour type. This mechanism-led comparison provides the framework for evaluating the event-defined emerging associations in Section 3.

### 2.6 DNA damage and genome instability as cross-cutting mechanisms

DNA tumour viruses can couple their replication or persistence to host DNA-damage pathways. HPV replication activates ATM- and ATR-associated responses, while E6/E7-mediated disruption of p53 and pRB weakens checkpoint control and promotes replication stress (Moody and Laimins, 2009; The Cancer Genome Atlas Research Network, 2017). HBV-related oxidative stress and integration can generate structural variation and dysregulate nearby host genes (Sung et al., 2012; Peneau et al., 2022). These processes can be viewed as a sequence in which viral replication or protein expression produces replication stress or DNA damage, defective checkpoint and repair responses permit mutation or rearrangement, and clonal selection retains advantageous host or virus–host events.

This framework also connects established mechanisms to the emerging associations discussed below. AAV2 and TTMV can generate selected insertion or fusion events, whereas lytic BKPyV infection can induce APOBEC3A and APOBEC3B and produce SBS2/SBS13-like damage in experimental urothelial models (Baker et al., 2022; Hatton et al., 2025). These observations support mechanistic hypotheses, but the relative contribution of virus-induced damage to tumour initiation must be evaluated separately for each association.

## 3 Recently recognised DNA virus–tumour associations

Although recently recognised DNA virus–tumour associations are heterogeneous, research has shifted from merely detecting viral sequences to defining specific virus–host events and their biological significance. TTMV::RARA represents a fusion gene, whereas HPV42-associated digital papillary adenocarcinoma exemplifies tissue-specific viral early-gene expression (Astolfi et al., 2021; Leiendecker et al., 2023). AAV2 insertion-positive HCC and transplant-associated BKPyV-positive urothelial carcinoma respectively exemplify cis-acting insertional activation and persistent T-antigen expression in the context of immunosuppression (Nault et al., 2015; Starrett et al., 2023). The principal value of these associations lies in helping to explain rare tumours or specific molecular subtypes, rather than in expanding viral screening in the general population.

To distinguish viral detection from a tumour-relevant event, we evaluated each emerging association across nine domains: frequency of the event; localisation to tumour cells; evidence of clonality; viral or virus–host transcription; replication in independent cohorts; functional evidence; evidence that suppression of the viral component reverses the malignant phenotype; event-matched diagnostic relevance; and current clinical development. Viral DNA detection alone was not considered equivalent to transcriptional activity or to a clonally retained virus–host event. We use a four-level working classification—established causal relationship, strongly supported emerging association, biologically plausible but incompletely validated association, and hypothesis-generating observation—to compare event-specific evidence rather than to assign a permanent status to an entire virus.

### 3.1 TTMV::RARA fusion-driven APL-like acute myeloid leukaemia

Torque teno mini viruses (TTMVs) are non-enveloped viruses with circular, negative-sense single-stranded DNA genomes classified within the family Anelloviridae. The human TTMV lineage discussed here belongs to the genus Betatorquevirus (International Committee on Taxonomy of Viruses, 2026). We retain TTMV as the clinically used abbreviation in TTMV::RARA. In this rare leukaemia, the tumour-relevant event is not an increased circulating viral load but insertion of TTMV DNA into RARA, which generates TTMV::RARA or, in some cases, RARA::TTMV::RARA transcripts (Astolfi et al., 2021; Tsai et al., 2024).

The fusion was first identified through transcriptome analysis of suspected APL cases (Astolfi et al., 2021). Conventional fusion analysis identified no known human RARA partner, whereas unmapped reads revealed junctions between TTMV sequences and RARA exons. Subsequent genome sequencing and breakpoint validation localised the viral insertion to RARA intron 2. Similar structures were then identified in independent cases (Zhou et al., 2025). A 2026 multicentre study of 25 patients further showed that distinct TTMV strains can participate in fusion formation and that all resolved insertions clustered in RARA intron 2 (Sun et al., 2026). A tripartite transcript comprising 5′ RARA, a TTMV segment and 3′ RARA was also detected in most transcriptome samples.

The biological effects of TTMV::RARA reflect coordinated changes in gene-expression control and protein function. The integrated segment contains an active viral promoter with tandem RUNX1-binding motifs that can enhance expression in myeloid cells. In the fusion protein, a virus-derived amino terminus replaces the regulatory region of native RARA, while the DNA-binding and ligand-binding domains are retained (Lou et al., 2025). The fusion protein can therefore enter the nucleus and participate in RARA/RXRA transcriptional complexes, but it alters retinoic acid-dependent regulation of target genes. Viral promoter-driven, myeloid-biased expression and abnormal RARA function jointly block granulocytic differentiation (Zhou et al., 2025; Sun et al., 2026). Leukaemic cells consequently exhibit an APL-like transcriptional profile that remains distinct from that of PML::RARA-positive APL. The proposed molecular mechanism of TTMV::RARA-associated leukaemia is summarised in **Figure 1**.

**Figure 1:**
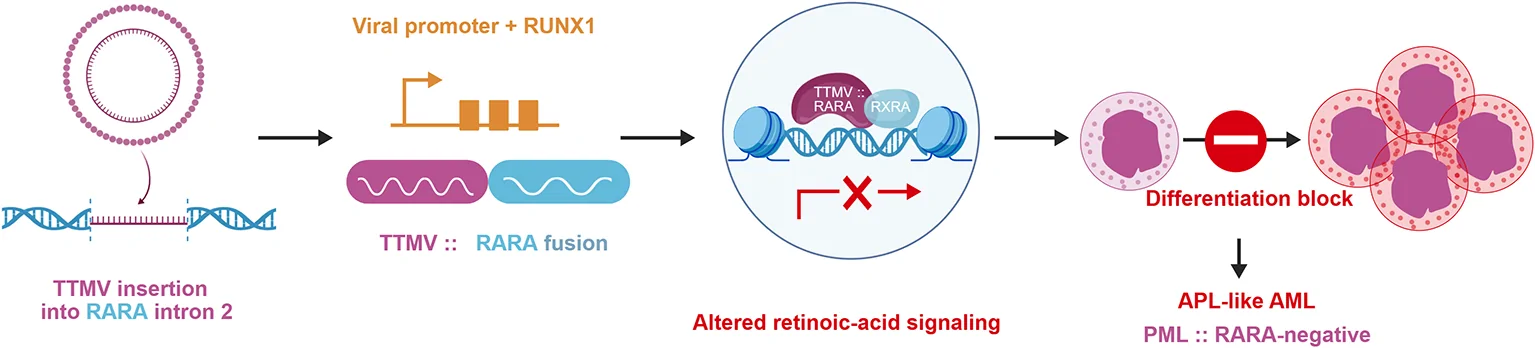
TTMV::RARA fusion in APL-like acute myeloid leukaemia. TTMV insertion into RARA intron 2 generates a fusion transcript that perturbs retinoic acid signalling and myeloid differentiation. The fusion junction, rather than viral load, is the defining diagnostic target.

Clinical data indicate that this disease predominantly affects children and adolescents. In the 25-patient cohort, 72% were aged 18 years or younger, and some had myeloid sarcoma or central nervous system involvement. Although most patients ultimately achieved complete remission, the first-course remission rate and long-term event-free survival were lower than in classic APL (Sun et al., 2026). Retention of the RARA ligand-binding domain in TTMV::RARA provides a biological rationale for treatment with all-trans retinoic acid (ATRA). A small retrospective cohort also suggested a potential therapeutic signal from combining ATRA with arsenic trioxide (Xu et al., 2025). However, no statistically conclusive differences among regimens have been shown, and the underlying mechanism and optimal combination strategy remain to be determined.

The discovery of TTMV::RARA extends the diagnostic pathway for APL-like leukaemia. Cases with APL morphology may test negative by PML::RARA fluorescence *in situ* hybridisation or common fusion assays. In such cases, RNA sequencing or fusion analysis should retain unmapped reads and include anellovirus sequences (Tsai et al., 2024; Zhou et al., 2025). Accurate identification can distinguish these patients from the broad category of “RARA-fusion-negative APL-like leukaemia” and provide a molecular basis for risk stratification and therapeutic studies (Sala-Torra et al., 2022). Patient-specific virus–host junctions may also support measurable residual disease monitoring, but the required assays and clinical thresholds remain to be established.

TTMV::RARA has been reproduced in independent reports and a multicentre series, and the recurrent RARA intron 2 insertion, fusion transcription and retained RARA functional domains support a driver role. However, the disease is rare, available clinical series are retrospective, direct suppression-and-rescue evidence for continued fusion dependence is limited, and treatment observations with ATRA or arsenic trioxide are not yet sufficient to define a standard regimen (Astolfi et al., 2021; Xu et al., 2025; Zhou et al., 2025; Sun et al., 2026).

### 3.2 HPV42 and digital papillary adenocarcinoma

Human papillomavirus type 42 (HPV42) is a non-enveloped virus with a circular double-stranded DNA genome. It has long been classified as a low-risk HPV type and is primarily associated with benign lesions such as warts. Recent studies have identified a strong association between HPV42 and digital papillary adenocarcinoma (DPA). DPA is a rare malignant cutaneous adnexal tumour that occurs mainly on the fingers and toes and can morphologically overlap with other sweat gland tumours. A 2023 study detected HPV42 in 45 of 47 DPA cases (Leiendecker et al., 2023). *In situ* detection, transcriptomic analysis and functional studies linked the viral signals to tumour cells. Subsequent independent pathology cohorts further supported this highly tissue-specific association.

DPA cells persistently express HPV42 E6 and E7, although no uniform pattern of viral integration is required. Some tumours contain integrated viral DNA, with breakpoints often disrupting E1 or E2 while retaining E6/E7. In others, viral genomes are maintained as extrachromosomal episomes (Kervarrec et al., 2023a; Leiendecker et al., 2023). Both configurations can support sustained early-gene transcription, indicating that integration is not the only prerequisite for the biological effects of HPV42 in DPA.

E7 accounts for much of the transforming activity attributed to HPV42. E7 binds pRB, RBL1 and RBL2, relieving their restraint on E2F transcription factors. This sustains the activity of genes involved in DNA replication and cell-cycle progression. E7 can also destabilised PTPN14, perturb Hippo–YAP signalling and suppress terminal epithelial differentiation (Leiendecker et al., 2023). These alterations accord with the marked expression of E2F target genes and a germ cell-like transcriptional programme in DPA. HPV42 E6 can bind E6AP and cooperate with E7 to extend the proliferative lifespan of primary cells. However, it degrades p53 less efficiently than HPV16 E6. Cell culture and xenograft experiments further showed that an intact HPV42 genome or E6/E7 expression can promote sustained proliferation and tumour formation (Leiendecker et al., 2023). The proposed tumour-promoting mechanisms of HPV42 in DPA are summarised in **Figure 2**.

**Figure 2:**
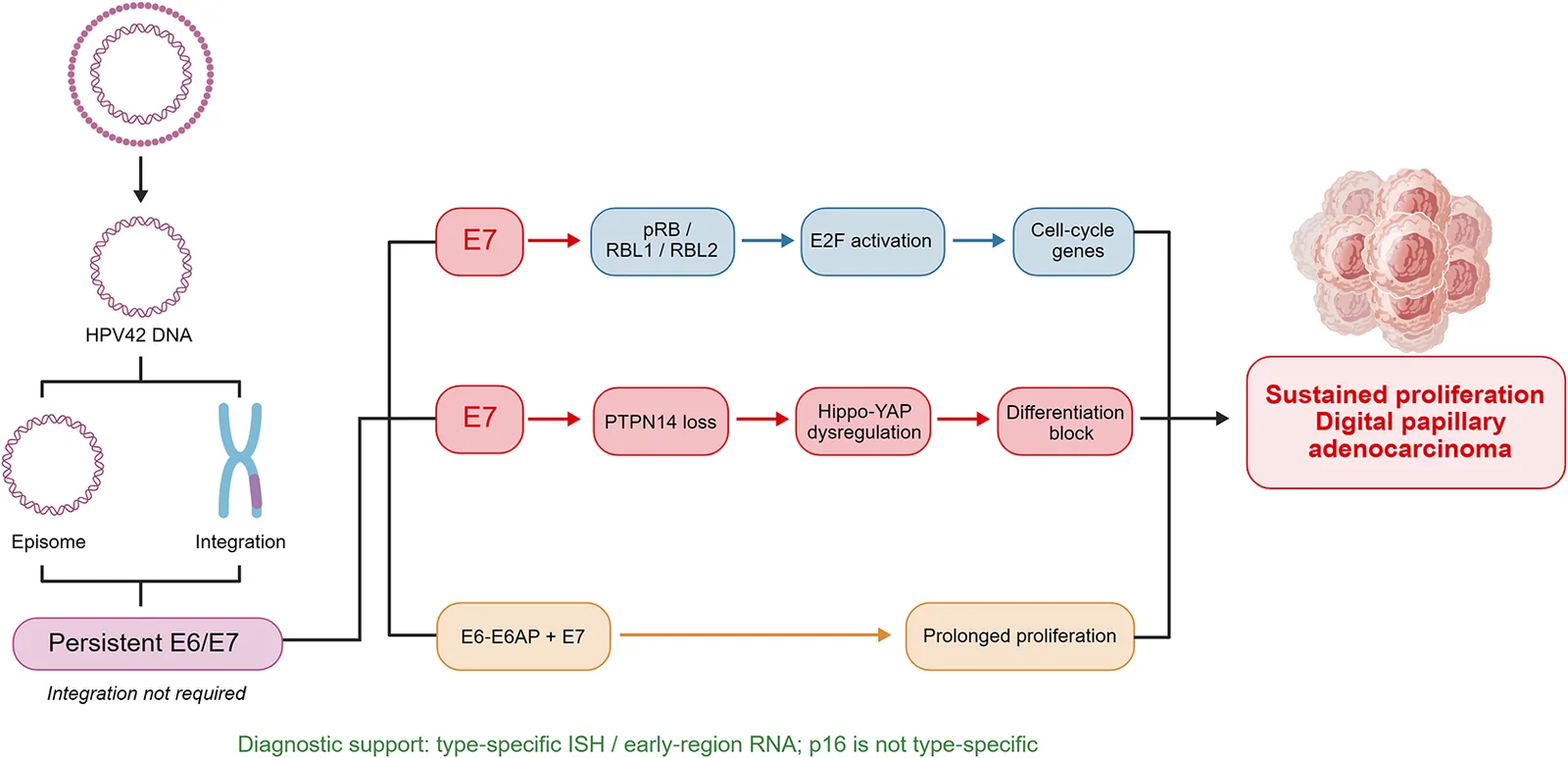
Proposed molecular model of HPV42-associated digital papillary adenocarcinoma. Episomal or integrated HPV42 may sustain E6/E7 expression, disrupting pRB–E2F and PTPN14–Hippo–YAP signalling. Tumour-cell HPV42 localisation or early-region RNA supports ancillary diagnosis; p16 alone is not specific.

HPV42 can serve as an ancillary molecular marker for DPA when interpreted together with morphology and anatomical context. The largest multi-platform study detected HPV42 in 45 of 47 DPA cases (96%) (Leiendecker et al., 2023). In an independent type-specific *in situ* hybridisation series, all 10 DPA cases were positive, whereas none of eight acral hidradenomas and none of 22 other sweat-gland tumours were positive (Vanderbilt et al., 2022). A later study using a pooled low-risk HPV probe rather than an HPV42-specific assay detected viral signal in six of 13 DPA patients and none of three acral hidradenomas, illustrating the potential effects of assay design, tissue quality and case classification (Chen et al., 2024). These data support type-specific HPV42 *in situ* hybridisation or early-region RNA detection as an ancillary test in morphologically equivocal lesions and in the evaluation of possible metastases. However, prospective diagnostic sensitivity and specificity have not been established, and a negative result alone does not exclude DPA. p16 overexpression lacks viral-type specificity and cannot replace tumour-cell localisation of HPV42 nucleic acids (Vanderbilt et al., 2022; Kervarrec et al., 2023b; Tekin et al., 2024; Goto, 2026).

HPV42 is highly enriched in DPA across independent cohorts, is localised to tumour cells, is transcriptionally active and has functional activity in cell and xenograft models. Nevertheless, DPA is rare, most series are retrospective, assay design affects detection, prospective diagnostic performance has not been established, and direct loss-of-function evidence for continued E6/E7 dependence remains incomplete (Vanderbilt et al., 2022; Leiendecker et al., 2023; Chen et al., 2024).

### 3.3 AAV2 insertions in a subset of hepatocellular carcinomas

Adeno-associated virus type 2 (AAV2), a member of the family *Parvoviridae*, is a non-enveloped virus with a linear single-stranded DNA genome. AAV2 generally requires helper functions supplied by adenoviruses or herpesviruses for efficient replication, whereas natural infection is common and usually asymptomatic. Its association with hepatocellular carcinoma (HCC) does not involve chronic viral hepatitis. Instead, the association involves insertion of wild-type AAV2 fragments into the host genome in a small subset of tumours, with sustained activation of growth-related host genes (Nault et al., 2015; La Bella et al., 2020).

A 2015 genomic and transcriptomic study of liver cancer first systematically identified AAV2–host junction sequences. Although insertion structures varied among tumours, recurrent sites involved genes including CCNA2, CCNE1, TERT, TNFSF10 and KMT2B (Nault et al., 2015; Bayard et al., 2018). These insertions were accompanied by overexpression of the corresponding genes or the production of virus–host chimeric transcripts. Most cases arose in non-cirrhotic livers, suggesting that low-frequency insertional events can occur outside the setting of conventional chronic liver disease. Subsequent studies observed similar target genes and transcriptional consequences in some cohorts, but detection has varied across regions and populations (Bayard et al., 2018; La Bella et al., 2020).

The key consequence of AAV2 insertion is not persistent viral protein production, but the use of viral regulatory elements to alter nearby host-gene expression. The 3′ regulatory region adjacent to the right-hand inverted terminal repeat has strong enhancer and promoter activity in hepatocytes (Tatsuno et al., 2019; La Bella et al., 2020). Even when the viral genome is incomplete, this region can drive transcription of neighbouring genes in either insertion orientation. When AAV2 inserts into CCNA2 or CCNE1, the viral promoter can splice to host exons, generating chimeric transcripts (Nault et al., 2015; Bayard et al., 2018)and directly increasing cyclin expression. Insertions near TERT can increase TERT expression, conferring a sustained proliferative advantage on abnormal clones. Local amplification, exon rearrangement and aberrant splicing can further enhance this cis-regulatory effect. The proposed insertion-driven mechanism of AAV2-associated HCC is summarised in **Figure 3**.

**Figure 3:**
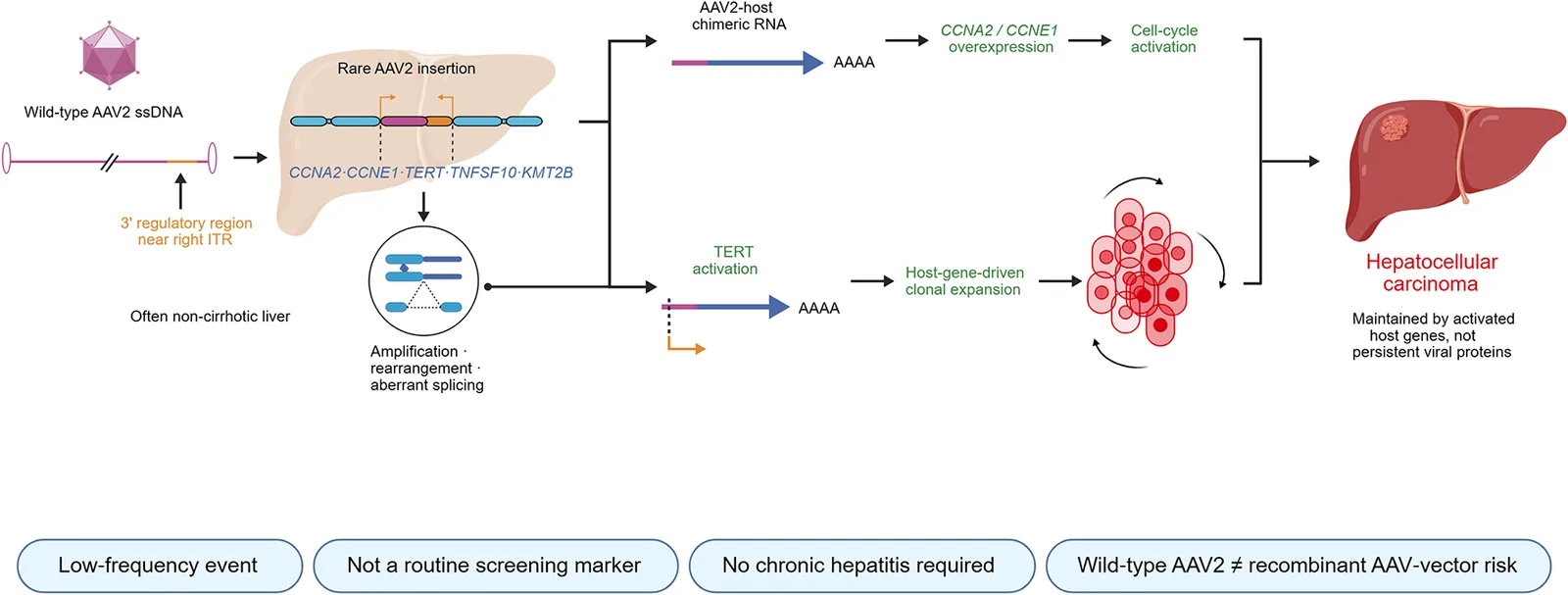
Host-gene activation by wild-type AAV2 insertion in hepatocellular carcinoma. Wild-type AAV2 insertion may generate chimeric transcripts or activate host genes such as CCNA2, CCNE1 and TERT. This rare event is not a routine screening marker and does not establish risk from recombinant AAV vectors.

This mechanism contrasts sharply with that of HBV-associated HCC. HBV contributes to liver tumour evolution through chronic inflammation, repeated regeneration, viral protein activity and widespread integration. AAV2 insertion-positive HCC, by contrast, is found in a subset of patients, often without evident cirrhosis. Subsequent tumour growth is maintained mainly by the activated host genes. AAV2 insertions are rare overall in HCC and are better regarded as a low-frequency molecular explanation for selected cases than as a general aetiology or screening marker (La Bella et al., 2020). Such analyses may be useful in research or diagnostically challenging cases but have not entered routine diagnosis. These observations derive from naturally acquired wild-type AAV2 and cannot be directly extrapolated to the oncogenic risk of recombinant AAV vectors (Logan et al., 2017; La Bella et al., 2020). The safety of gene therapy vectors should be assessed independently based on vector design, dose, integration profile, and long-term follow-up data.

Recurrent clonal AAV2 insertions with local host-gene activation support insertional oncogenesis in a small subset of HCC. However, event frequency varies across cohorts and regions, continued viral transcription or protein dependence is not expected, and findings from naturally acquired wild-type AAV2 cannot be extrapolated to recombinant AAV vectors. AAV2 insertion should therefore remain a research-level, low-frequency molecular explanation for selected HCCs (Nault et al., 2015; Logan et al., 2017; La Bella et al., 2020).

### 3.4 Transplant-associated BKPyV-positive urothelial carcinoma

BK polyomavirus (BKPyV) is a non-enveloped virus with a circular double-stranded DNA genome. Its early region encodes the large T antigen (LTag) and small T antigen (sT). Primary infection usually occurs during childhood, after which the virus can persist in the kidneys and urothelium. Immunosuppression following organ or haematopoietic stem cell transplantation can promote viral reactivation, leading to BKPyV-associated nephropathy or haemorrhagic cystitis (Kotton et al., 2024). Molecular links between BKPyV and high-grade urothelial carcinoma have so far been reported mainly in transplant-associated cases involving prolonged immunosuppression (Bertz et al., 2020; Starrett et al., 2023).

This association was initially suggested by pathological observations in urothelial carcinomas arising in transplant recipients. Some tumours showed diffuse, strong nuclear T-antigen staining. Targeted sequencing and whole-genome analysis subsequently identified BKPyV–host junctions and reconstructed multicopy, rearranged or tandem integration structures (Wang et al., 2020). A 2023 analysis of bladder cancers from 43 solid-organ transplant recipients identified nine BKPyV-positive tumours (Starrett et al., 2023). Several showed viral integration, viral transcription and LTag expression. In some cases, matched primary and metastatic lesions shared viral integration sites but also harboured non-shared sites (Bertz et al., 2020; Starrett et al., 2023). This pattern suggests that early clonal events may coexist with subsequent structural heterogeneity.

BKPyV integration is often accompanied by structural selection that favours cellular transformation but compromises lytic replication. The late region or VP1 is disrupted, limiting virion production, whereas the T-antigen-encoding early region is retained and persistently transcribed (Starrett et al., 2023). LTag binds pRB family proteins and relieves their repression of E2F, while also interfering with the p53-mediated DNA damage response. sT can modulate PP2A-related signalling (Starrett et al., 2023), further affecting cell proliferation and survival. Some cohorts suggest that persistent T-antigen expression may functionally substitute for a subset of RB/p53 pathway alterations. However, host mutational profiles have not been fully consistent across studies.

Beyond integration and persistent T-antigen expression, primary urothelial cells and organ-culture models suggest that lytic BKPyV infection can activate interferon responses and induce APOBEC3A and APOBEC3B (Baker et al., 2022; Hatton et al., 2025). While restricting viral replication, these cytidine deaminases may also generate host-genome mutational patterns resembling SBS2 and SBS13 (Baker et al., 2022; Hatton et al., 2025). This mechanism offers an alternative explanation for how BKPyV may affect urothelial cells, although its contribution to bladder cancer in the general population remains uncertain. A 2026 study of 19 kidney transplant-associated urothelial carcinomas identified BKPyV integration in six cases (Wang et al., 2026). The study also suggested that BKPyV integration and aristolochic acid-associated alterations may represent distinct molecular pathways. These findings further indicate that transplant-associated urothelial carcinoma is not a single biological entity.

For urothelial tumours arising in transplant recipients, tumour-cell T-antigen immunoreactivity can prompt targeted viral testing. A credible BKPyV attribution should combine T-antigen expression with BKPyV-specific DNA or RNA detection in tumour tissue, preferably with localisation of the viral signal to tumour cells. Integration analysis provides strong evidence of clonal viral involvement and is recommended where technically feasible, but it should not be treated as an obligatory diagnostic criterion because integration is not demonstrable in every BKPyV-positive tumour. Commonly used antibodies against SV40 large T antigen cross-react with several polyomaviruses and cannot establish the viral type on their own (Starrett et al., 2023; Wang et al., 2023, 2026).

Monitoring of BKPyV viraemia, nephropathy or haemorrhagic cystitis primarily serves the management of transplant-associated viral disease; current evidence does not support viraemia as a screening marker for urothelial carcinoma (Kotton et al., 2024). Persistently expressed LTag remains a hypothetical or preclinical therapeutic target, and direct clinical evidence is lacking. The proposed pathways are summarised in **Figure 4**.

**Figure 4:**
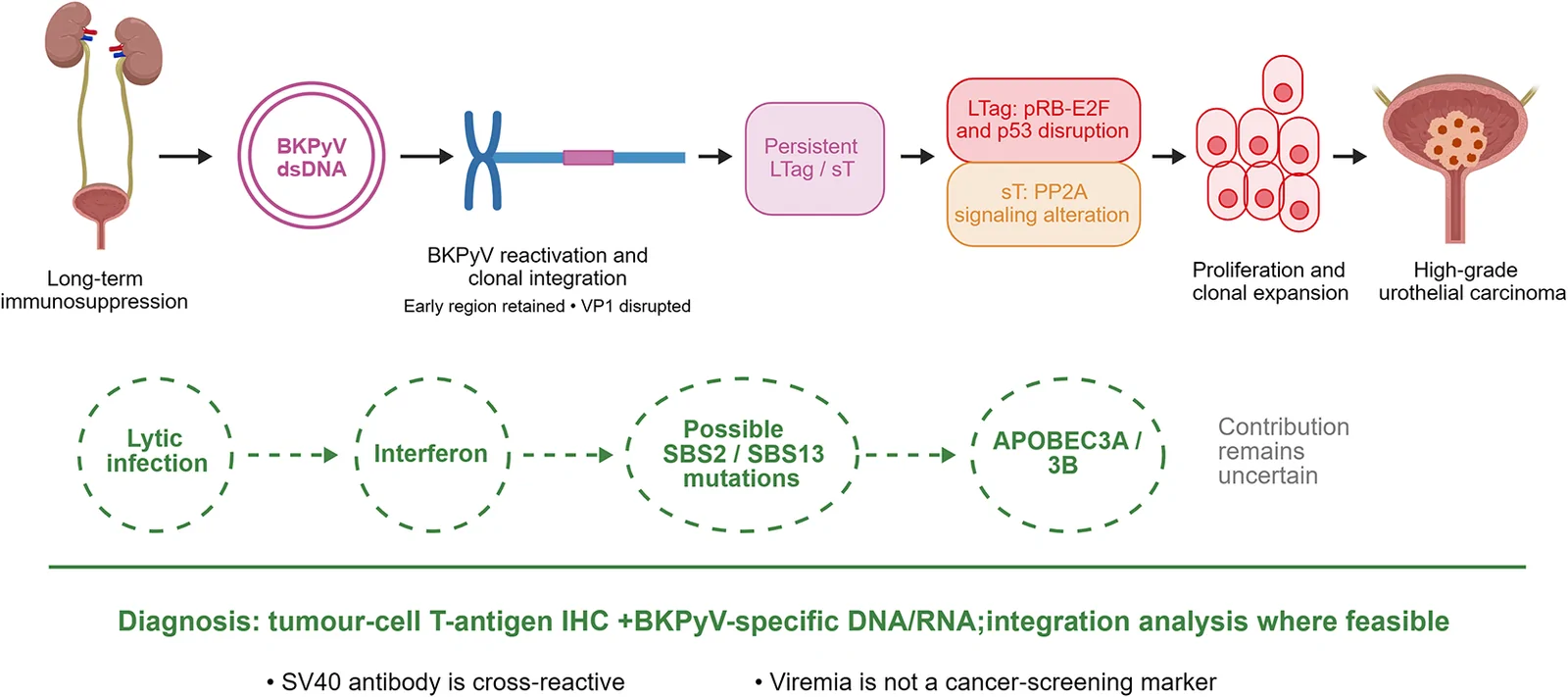
Proposed model of BKPyV-positive urothelial carcinoma in immunosuppressed patients. BKPyV integration and persistent T-antigen expression may disrupt pRB–E2F/p53 and PP2A signalling; the interferon–APOBEC pathway remains provisional. Tumour attribution combines T-antigen immunohistochemistry with BKPyV-specific DNA/RNA testing, with integration analysis where feasible.

BKPyV-specific nucleic acids, clonal integration and T-antigen expression support a biologically relevant association in a subset of transplant-associated urothelial carcinomas. The evidence is nevertheless concentrated in immunosuppressed populations; integration and T-antigen expression are heterogeneous; commonly used T-antigen antibodies are not virus-specific; prospective prevalence and diagnostic specificity are unknown; and direct evidence of continued tumour dependence on BKPyV proteins is lacking. Relevance to urothelial carcinoma in the general population remains uncertain (Bertz et al., 2020; Starrett et al., 2023; Wang et al., 2026).

In all four associations, tumours retain distinct virus–host events that can be resolved molecularly, but these events differ in how they sustain the malignant phenotype. TTMV::RARA-associated tumours centre on persistent expression of the fusion product, and HPV42-associated tumours on persistent expression of early viral proteins (Astolfi et al., 2021; Leiendecker et al., 2023). In BKPyV-positive urothelial carcinoma, T-antigen expression following integration may contribute to tumour maintenance together with alterations in host pathways (Starrett et al., 2023). AAV2 insertion acts mainly through cis activation of host genes (Nault et al., 2015; La Bella et al., 2020), whereas subsequent tumour growth may not require continued viral gene expression. Accordingly, these mechanistic differences shape the choice of assay targets, therapeutic targets and routes to clinical translation. The associated tumour, retained molecular event, evidence strength and present clinical development stage of the four emerging associations are compared directly in **Table 2**.

**Table 2:** Evidence strength and clinical maturity of four emerging virus-tumour associations.

Evidence dimension	TTMV::RARA	HPV42	AAV2	BKPyV
Associated tumour and context	APL-like AML lacking canonical RARA fusions	Digital papillary adenocarcinoma (DPA), including non-acral cases	A low-frequency subset of HCC, often arising in non-cirrhotic liver	High-grade urothelial carcinoma, reported mainly in immunosuppressed transplant recipients
Defining retained viral event	TTMV insertion into RARA intron 2, producing TTMV::RARA or RARA::TTMV::RARA transcripts	Tumour-retained episomal or integrated HPV42 with persistent early-region (E6/E7) transcription; integration is not required	Clonal wild-type AAV2 insertion near CCNA2, CCNE1, TERT, TNFSF10 or KMT2B, with cis activation or chimeric transcription	Early-region retention, clonal integration in a subset, and variable persistent large T-antigen expression
Key cohort evidence	10/2,553 screened AML cases in the 2026 multicentre analysis; 25 cases in the integrated dataset (Sun et al., 2026)	45/47 DPA in the largest study; type-specific ISH: 10/10 DPA versus 0/8 acral hidradenomas and 0/22 other sweat-gland tumours. A separate pooled low-risk HPV probe study detected 6/13 DPA patients versus 0/3 acral hidradenomas; this assay was not HPV42-specific (Vanderbilt et al., 2022; Leiendecker et al., 2023; Chen et al., 2024)	11/193 HCCs in the discovery cohort; prevalence varies across cohorts and regions (Nault et al., 2015)	BKPyV in 9/43 post-solid-organ-transplant bladder cancers; integration in 6/19 kidney-transplant-associated urothelial carcinomas (Starrett et al., 2023; Wang et al., 2026)
Tumour-cell localisation, clonality and transcription	Recurrent intron 2 junctions and fusion RNA; tripartite RARA::TTMV::RARA transcripts in 13/15 evaluable transcriptomes	Tumour-cell DNA/RNA localisation and early-gene expression; oncogenic evidence does not depend on integration	Tumour-specific junctions, chimeric transcripts and increased expression of nearby host genes	BKPyV-specific DNA/RNA, tumour-cell T-antigen expression and clonal integration in a subset; expression is heterogeneous
Functional and continued-dependence evidence	Retained RARA DNA- and ligand-binding domains, viral promoter/RUNX1 motifs, altered RARA/RXRA programmes and differentiation blockade. Persistent fusion expression is implicated; direct suppression-and-rescue evidence remains limited	E7 perturbs pRB-family proteins and PTPN14-Hippo-YAP signalling; E6/E7 support proliferation and xenograft growth. Direct loss-of-function evidence for continued tumour dependence remains incomplete	Viral regulatory elements activate host genes in cis. Continued tumour maintenance is expected to be host-gene driven rather than dependent on persistent viral protein	LTag perturbs pRB/p53 pathways; sT can affect PP2A-related signalling, and lytic infection can induce APOBEC enzymes in experimental models. Direct tumour dependence is not established
Independent replication and principal limitation	Independent cases followed by multicentre validation; rarity and retrospective treatment/functional evidence remain limiting	Independent molecular-pathology cohorts support enrichment and tumour-cell localisation; prospective diagnostic validation and direct dependency evidence are lacking	Similar insertion targets have been reported independently, but frequency varies by cohort and region; natural wild-type AAV2 findings should not be extrapolated directly to recombinant AAV vectors	Multiple transplant-associated series report BKPyV-related events, but assays and findings are heterogeneous; prospective specificity and direct dependence remain unresolved
Diagnostic relevance and clinical stage	Specialist molecular classification using RNA sequencing or fusion analysis that retains unmapped reads; patient-specific junction assays for residual disease remain investigational	Type-specific HPV42 ISH or early-region RNA assays may support specialist ancillary diagnosis; p16 is not specific, and prospective validation is absent	Insertion-site mapping plus target-gene expression analysis by WGS, capture sequencing or long-read methods; research-stage attribution only	Tumour-cell T-antigen expression plus BKPyV-specific DNA/RNA; integration analysis strengthens attribution where technically feasible. Context-restricted specialist/research use; no population-screening role
Therapeutic relevance and overall evidence maturity	ATRA/ATO has biological rationale, but no standard regimen is established. Overall: strongly supported emerging driver event	E6/E7 are research targets; no validated virus-directed treatment. Overall: strongly supported emerging association with functional oncogenic evidence	No current rationale for AAV2-directed therapy once maintenance is host-gene driven. Overall: biologically plausible, low-frequency insertional driver	LTag is a hypothetical target; treatment follows urothelial carcinoma type, stage and patient context. Overall: biologically plausible but incompletely validated association

## 4 Prevention, diagnosis and treatment of DNA virus-associated tumours

Research on classical DNA tumour viruses has yielded established or relatively mature clinical applications, including vaccination, antiviral therapy, pathology-based biomarkers and viral-load monitoring (Chan et al., 2017; Lei et al., 2020; European Association for the Study of the Liver, 2025). Clinical implications are considered here in four categories: established clinical practice; clinically promising or specialist applications; research-stage diagnostic approaches; and hypothetical therapeutic targets. This distinction is important because a biologically plausible viral target does not by itself establish clinical utility.

### 4.1 Prevention

Vaccination against HPV and HBV is the most established strategy for preventing virus-associated cancers (Chang et al., 1997; Lei et al., 2020). When administered before infection, HPV vaccines substantially reduce persistent infection with vaccine-covered types and the associated precancerous lesions. However, current vaccines do not cover all oncogenic HPV types, and cervical cancer screening should continue after vaccination according to age and individual risk. HBV control requires timely neonatal vaccination, prevention of mother-to-child transmission, blood safety and safe medical practices. For individuals with chronic HBV infection, nucleos(t)ide analogues can provide long-term suppression of viral replication and reduce the risks of cirrhosis and HCC. However, they do not completely eliminate cccDNA, pre-existing viral integrations or abnormal hepatocyte clones, and high-risk patients therefore still require HCC surveillance (European Association for the Study of the Liver, 2025).

EBV, KSHV and BKPyV currently lack established vaccines for cancer prevention, so control efforts focus mainly on immunocompromised populations. Monitoring of EBV and BKPyV in transplant recipients should account for transplant type, donor and recipient status, immunosuppression intensity and clinical presentation (Kotton et al., 2024; Preiksaitis et al., 2024). In people living with HIV, sustained effective antiretroviral therapy can restore immune function and reduce the risk of KSHV-associated Kaposi sarcoma. TTMV::RARA, HPV42-associated DPA and AAV2 integration are low-frequency molecular events in specific tumour settings and are not currently suitable for population-wide screening (La Bella et al., 2020; Astolfi et al., 2021; Leiendecker et al., 2023). BKPyV monitoring primarily serves the management of transplant-associated viral disease, and its value in predicting urothelial carcinoma risk has not been established.

### 4.2 Molecular diagnosis and disease monitoring

Several biomarkers of classical tumour viruses have entered clinical workflows for tumour diagnosis or disease monitoring. In cervical screening, high-risk HPV positivity primarily signals viral persistence and the risk of precancerous lesions. It must therefore be interpreted together with cytology, triage testing or tissue biopsy. p16 overexpression can serve as a surrogate marker for some HPV-associated tumours, although its applicability depends on anatomical site and histopathological type. EBER *in situ* hybridisation can identify cells harbouring latent EBV infection, whereas LANA immunohistochemistry can identify KSHV-associated tumours (Weiss et al., 1987; Ballestas et al., 1999). MCPyV-associated MCC can be assessed by combining T-antigen expression with viral nucleic acid detection (Houben et al., 2010). Plasma EBV DNA is used to monitor treatment and assess recurrence in nasopharyngeal carcinoma and has been evaluated for screening in high-incidence regions (Chan et al., 2017). In HPV-associated oropharyngeal cancer, circulating tumour HPV DNA in plasma has also shown potential for recurrence monitoring (Chera et al., 2020). However, its use remains dependent on the assay platform, sampling time and clinical context.

For recently recognised associations, testing should move from asking whether a virus is present to identifying a defining virus–host event. Detection of TTMV::RARA requires RNA sequencing or fusion analysis capable of identifying virus–host junctions in APL-like cases lacking common RARA fusions (Tsai et al., 2024; Zhou et al., 2025). HPV42-associated DPA requires type-specific *in situ* detection or early-region RNA assays to localise the viral signal to tumour cells (Vanderbilt et al., 2022). In both settings, diagnostic value derives from defining a specific molecular subtype or pathological entity rather than detecting a general increase in viral load.

Evaluation of a suspected BKPyV-associated urothelial carcinoma should combine tumour-cell T-antigen expression with BKPyV-specific DNA or RNA detection. Integration analysis should be used where technically feasible to strengthen evidence of clonal viral involvement and define viral–host structure, but its absence or technical unavailability does not by itself exclude the association. Commonly used antibodies against SV40 large T antigen cross-react with several polyomaviruses and cannot establish the viral type on their own. Analysis of AAV2-associated HCC instead focuses on intratumoural insertion structures and dysregulation of nearby genes such as CCNA2, CCNE1 and TERT (Nault et al., 2015; Bayard et al., 2018). BKPyV load in blood or urine is used mainly to manage transplant-associated viral disease, whereas AAV2 insertion testing remains a research approach. Neither has an established role in general-population screening.

### 4.3 Treatment

Once a virus-associated tumour has been diagnosed, controlling viral infection and treating the established tumour should be considered separately. Antiviral drugs are used primarily to suppress replication, reduce inflammation or prevent reactivation. Established tumours should still be treated according to histopathological type, stage and patient status, using surgery, radiotherapy, chemotherapy, immunotherapy or other systemic treatments (Preiksaitis et al., 2024; European Association for the Study of the Liver, 2025). When a virus acts only as an initial trigger of host-gene dysregulation, reducing the burden of free virus generally does not induce regression of the established tumour.

Host immune status is closely linked to the treatment of several virus-associated tumours. HIV-associated Kaposi sarcoma first requires sustained effective antiretroviral therapy, although patients with advanced disease still require local or systemic treatment. Depending on disease severity, EBV-associated post-transplant lymphoproliferative disease may be managed by reducing immunosuppression, anti-CD20 therapy, chemotherapy or virus-specific T-cell therapy (Trappe et al., 2012; Mahadeo et al., 2024; Toh et al., 2024). Immune checkpoint inhibitors are used for advanced MCC (Kaufman et al., 2016; Nghiem et al., 2016), but treatment selection should remain based on tumour type and clinical stage rather than viral status alone.

Retention of the RARA ligand-binding domain in TTMV::RARA provides a biological rationale for ATRA treatment (Xu et al., 2025; Zhou et al., 2025). Responses to ATRA, arsenic trioxide and chemotherapy have varied across retrospective reports, however, and regimens developed for PML::RARA-positive APL cannot yet be adopted as standard treatment for this molecular subtype.

For HPV-related disease, therapeutic vaccination remains investigational; Vvax001 has shown activity in HPV16-positive high-grade cervical intraepithelial neoplasia rather than establishing a standard treatment for invasive cancer (Eerkens et al., 2025). For MCPyV-positive MCC, virus-specific T-cell therapy has produced clinical responses in small studies, but it is not established standard care (Chapuis et al., 2014). HPV42 E6/E7 and BKPyV LTag remain hypothetical or preclinical therapeutic targets. By contrast, AAV2-positive HCC offers no rationale for therapy directed against AAV2 because tumour maintenance appears to depend mainly on host-gene dysregulation.

## 5 Discussion and outlook

Classical oncogenic DNA viruses and recently recognised associations are not simply old and new versions of the same problem. They represent different scales of disease and distinct research priorities. High-risk HPV, HBV, EBV, KSHV and MCPyV are associated with substantial disease burdens. Their oncogenic mechanisms are comparatively well characterised, and some clinical interventions are established. By contrast, recently recognised associations are concentrated in rare tumours or specific molecular subtypes. Their value lies chiefly in providing molecular definitions for cases that were previously difficult to explain. Accordingly, a new association should not be judged solely by its viral detection rate. Evaluation should establish whether a specific virus–host event can account for the tumour phenotype and whether it is retained or transcriptionally active in tumour cells. It should also determine whether identifying the event can alter diagnosis or clinical management (Nault et al., 2015; Astolfi et al., 2021; Leiendecker et al., 2023; Starrett et al., 2023).

On this basis, TTMV::RARA-positive APL-like AML and HPV42-associated DPA are classified here as strongly supported emerging associations because the defining events have been reproduced, localised to tumour cells and linked to plausible functional consequences. AAV2 insertion-positive HCC is best considered a biologically plausible, low-frequency insertional driver in selected tumours because the retained consequence is host-gene dysregulation rather than ongoing viral-protein dependence. BKPyV-positive urothelial carcinoma is likewise biologically plausible but incompletely validated: the strongest evidence is concentrated in transplant recipients, and integration and T-antigen expression are heterogeneous. These labels describe the present evidence for the specific event–tumour pairing and do not imply equal population burden, clinical readiness or formal carcinogen classification.

Mechanistically, the four emerging associations form a continuum from persistent viral products to host-genomic tumour maintenance. HPV42-associated DPA lies near persistent viral early-protein expression, whereas TTMV::RARA-positive APL-like AML is sustained by a virus–host fusion transcript (Leiendecker et al., 2023; Zhou et al., 2025). BKPyV-positive urothelial carcinoma occupies an intermediate position in which variable T-antigen expression, integration, host-pathway alterations and immunosuppression may coexist (Starrett et al., 2023). AAV2 insertion-positive HCC lies near the host-driven end because viral regulatory elements activate nearby genes in cis and persistent viral-protein expression is not expected (Nault et al., 2015; La Bella et al., 2020). This mechanistic continuum is summarised in **Figure 5**. Its purpose is to organise diagnostic and therapeutic implications, not to rank causal certainty or clinical readiness.

**Figure 5:**
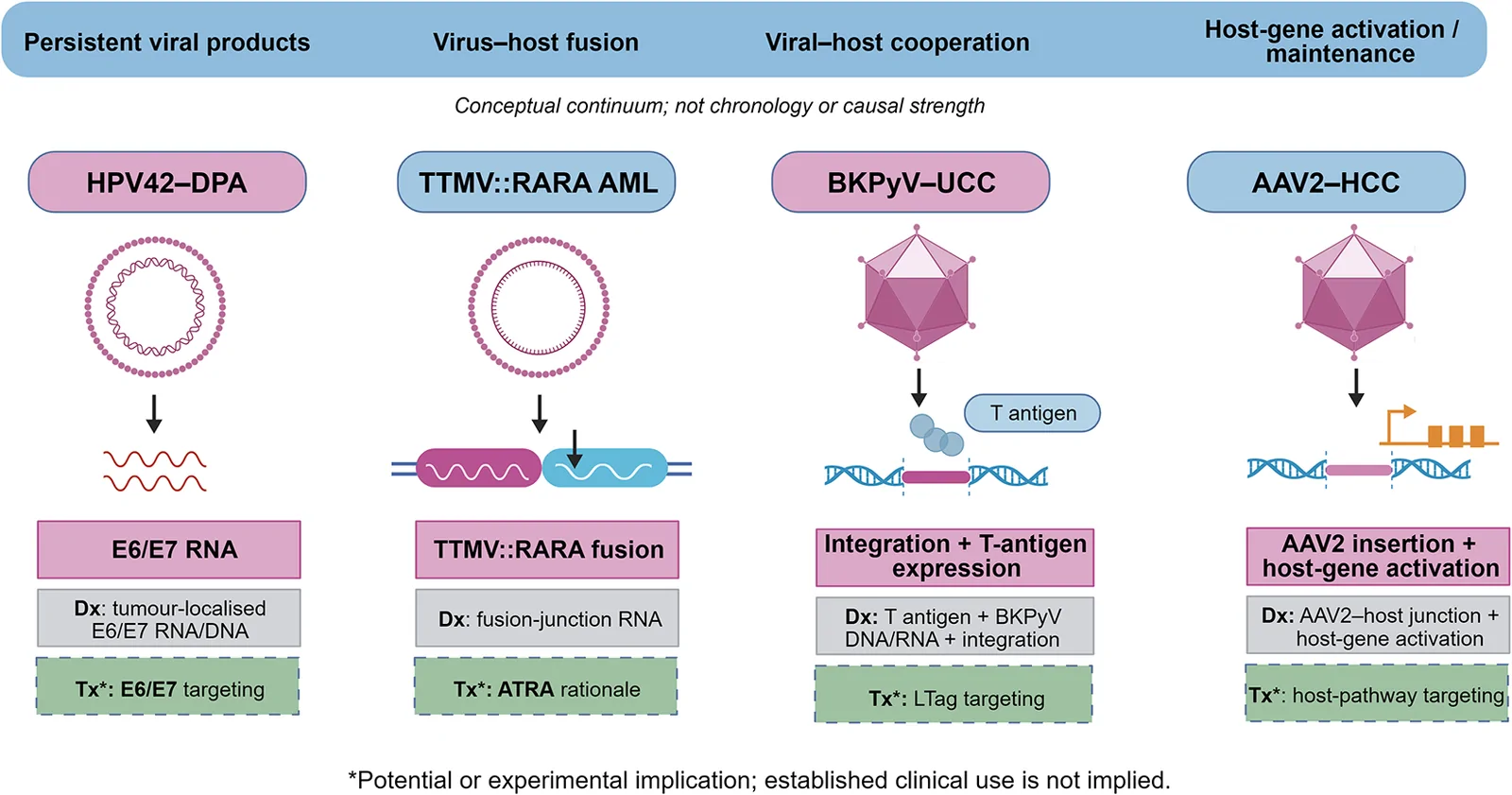
Tumour-retained viral events and their diagnostic and therapeutic implications. Four emerging associations span a continuum from persistent viral-product expression to host-gene activation. Each module links the retained event to its diagnostic target and potential therapeutic implication; positions do not indicate causal strength or clinical readiness.

Tissue and host context also determine whether a viral event gains a selective advantage. The marked enrichment of HPV42 in DPA indicates that existing “high-risk” and “low-risk” classifications cannot be extrapolated uncritically across tissues (Vanderbilt et al., 2022; Leiendecker et al., 2023). BKPyV-associated urothelial carcinoma occurs mainly in transplant recipients under prolonged immunosuppression (Starrett et al., 2023; Wang et al., 2026). This pattern suggests that viral reactivation, weakened immune surveillance and host carcinogenic exposures may together shape the observed spectrum of cases. AAV2 insertion-positive HCC has been reported mainly in a subset of cases without overt cirrhosis. However, its low frequency and geographical variation limit broader extrapolation. Recently recognised associations are therefore better understood as specific tumour subtypes arising from viral type, host-cell state and selectively retained molecular events.

Discovery and validation methods should be matched to the event under investigation. RNA fusion analysis and examination of unmapped reads can identify TTMV::RARA (Tsai et al., 2024). Type-specific *in situ* and transcriptional assays can establish the cellular localisation of HPV42 signals (Vanderbilt et al., 2022). Whole-genome and long-read sequencing are suited to reconstructing complex AAV2 or BKPyV insertion structures. Unbiased viral searches can nominate candidate associations but cannot replace *in situ* localisation, junction-site validation, analysis of transcriptional consequences or functional studies (Zapatka et al., 2020; Leiendecker et al., 2023). Integrating matched tumour and normal tissues, paired primary tumours and metastases, and longitudinal samples can help distinguish clonal tumour events, bystander infection and viral reactivation under immunosuppression. Where feasible, single-cell or spatial analyses may further aid this distinction.

Computational viral genomics can complement read-level detection by interrogating sequence constraints and virus–host evolutionary relationships. One emerging example is the analysis of relative minimal absent words, which are short sequences present in a viral genome but absent from the reference host genome. Using the EAGLE algorithm and the GRCh38 human reference genome, Georgakilas et al. identified highly conserved 11- and 12-bp prevalent relative minimal absent words in EBV, HBV, HTLV-1 and HHV-8/KSHV, whereas comparable short motifs were not detected in HPV or HCV. The motifs showed non-random localisation within viral genes involved in replication, persistence, immune modulation or oncogenic signalling (Georgakilas et al., 2026). Such analyses may generate hypotheses concerning viral sequence constraints and long-term host adaptation that are not captured by presence-or-absence testing. Their biological interpretation, tumour specificity and diagnostic performance require independent validation, however, and the approach remains exploratory rather than clinically established.

Current evidence remains limited by small case numbers, retrospective enrichment, geographical and population differences, non-standardised testing workflows and insufficient validation of functional dependence (La Bella et al., 2020; Vanderbilt et al., 2022; Sun et al., 2026; Wang et al., 2026). Near-term translation should therefore focus on clearly defined clinical gaps: molecular diagnosis of APL-like leukaemia and DPA, targeted BKPyV validation in urothelial carcinomas from transplant recipients, and research-only attribution of AAV2 insertions. Multicentre prospective registries should standardise sampling and testing, report negative results, and compare new assays with routine pathological and molecular workflows.

At the population level, control of virus-associated cancer should continue to prioritise HPV and HBV vaccination, chronic infection management and surveillance of high-risk populations (Chang et al., 1997; Lei et al., 2020; European Association for the Study of the Liver, 2025). Precision research on low-frequency associations should complement, rather than dilute, these validated public-health strategies.

## 6 Conclusion

DNA viruses can contribute to tumour development through persistent viral oncoprotein expression, latency-associated gene programmes and chronic tissue damage, as well as through specific virus–host events. These events include fusion genes, tissue-specific viral early-gene expression and low-frequency insertional activation. Studies of TTMV::RARA, HPV42, AAV2 and BKPyV have broadened the recognised spectrum of DNA virus-associated cancers. However, the strength of the evidence, mechanisms of tumour maintenance and clinical relevance differ among these associations.

The next priority is not simply to expand the catalogue of virus-positive tumours. Event-matched methods should confirm a viral event in tumour cells and define its structure, expression, clonality and functional consequence. Clinical utility should then be evaluated prospectively in settings in which detection could alter diagnosis or management. For the four emerging associations, these steps should precede population screening or virus-directed therapeutic claims.
